# Study on the motor development and biomechanical characteristics of children aged 3–5 years

**DOI:** 10.3389/fpubh.2025.1525824

**Published:** 2025-09-08

**Authors:** Bojie Hou, Jie Zhao, Zhongqiu Ji, Guiping Jiang, Zhanbing Song

**Affiliations:** ^1^College of Physical Education and Sport Science, Beijing Normal University, Beijing, China; ^2^Sport Coaching College, Beijing Sport University, Beijing, China; ^3^College of Physical Education and Health Science, Zhejiang Normal University, Jinhua, China

**Keywords:** gait, coordination pattern, preschool children, child development, human simulation

## Abstract

**Objective:**

This study analyzes changes in gait biomechanics in children aged 3–5, exploring motor development patterns during this critical period.

**Methods:**

Using the BTS SMART DX infrared system and Kistler 3D force plate, three-dimensional gait motion and ground reaction forces were collected from 3-, 4-, and 5-year-olds during walking. Inverse dynamics analysis with Anybody 7.4 software provided detailed joint moments, muscle forces, and joint angles. Coordination patterns of joint angles and moments, Lyapunov exponents, muscle force data, joint energy absorption, and power were further analyzed.

**Results:**

Joint angle coordination patterns remained consistent across ages, while joint moment control patterns simplified from three to two with age, indicating progressive joint control development. Muscle strength, joint power, and gait stability improved with age, reflecting enhanced movement efficiency and adaptability to complex motor tasks.

**Conclusion:**

Gait control in 3-year-olds is immature and mainly hip- and knee-dependent. At 4 years, children show significant joint coordination changes with increased ankle involvement, marking a transitional phase. By age 5, children exhibit more complex and stable gait control, though still developing. Overall, gait stability and coordination increase with age, with 4 years as a critical developmental period and 5 years showing more refined control characteristics.

## 1 Introduction

Motor development in early childhood represents a fundamental indicator of the maturation of the nervous and motor systems. The preschool period (approximately 3–5 years of age) constitutes a critical window for motor development, during which acquisition of motor skills not only affects children’s daily functional abilities but also exerts lasting influence on subsequent motor learning and broader developmental outcomes (1, 2). Nervous system maturation contributes substantially to motor development by improving temporal and spatial coordination of muscle activation and inter-segmental joint motion, which are prerequisites for stable and controlled gait performance. Consequently, gait—frequently adopted as a core metric of motor maturation—provides an observable index of balance, coordination, and dynamic motor characteristics across development (3). Moreover, gait metrics are closely associated with later physical activity capacity and overall health status (4, 5).

During early childhood, gait patterns undergo marked transformation, evolving from relatively unstable and uncoordinated locomotion toward more mature, adult-like patterns (6). Empirical evidence indicates that certain adult-like features, such as reciprocal arm swing, may emerge by approximately 3.5 years of age (7). With increasing age, gait parameters such as stride length, cadence, and gait symmetry are progressively refined, with a mature gait pattern typically established by the age of 7 (8). These developmental changes reflect neurodevelopmental refinements in muscle coordination that produce smoother and more stable locomotor behavior; concurrently, the dynamic properties of lower limb joints also mature during this interval (9).

Although walking is a basic human behavior, its physiological control is complex, necessitating coordinated contributions from multiple joints and musculature. Effective locomotion requires the motor system to manage the high number of mechanical degrees of freedom (DoF), often by constraining coordination through consistent weighting patterns among joints and muscles to achieve whole-body coordination (10–12). This coordination evolves with neuromuscular maturation, which must entrain and synchronize muscle activation patterns to sustain stable gait. Accordingly, the development of dynamic balance during locomotion is widely regarded as a salient marker of gait maturity (13). Gait stability is operationalized as the ability of the locomotor system to maintain or restore its state in response to external perturbations (14); therefore, assessment of age-related differences in joint-level stability is central to evaluating gait maturity and motor development.

Contemporary gait analysis employs techniques such as Principal Component Analysis (PCA) and Lyapunov Exponent (LyE) to elucidate control strategies and stability properties. PCA facilitates identification of dominant movement patterns, thereby clarifying how components of neuromuscular coordination contribute to global gait behavior (15). The LyE quantifies local dynamic stability by estimating the divergence rate of reconstructed state-space trajectories derived from kinematic time series; larger LyE values denote greater sensitivity to perturbation and reduced stability. Thus, LyE has been applied extensively to characterize stability and fall risk in locomotion (16–18). These analytical approaches yield complementary perspectives on the relationships among neuromuscular control, dynamic stability, and developmental progression of motor function in children.

To mitigate limitations inherent to any single analytical approach, the present study integrates Lyapunov Exponent (LyE) and Principal Component Analysis (PCA) with complementary biomechanical indices, specifically lower-limb muscle strength, joint power, and the energy-absorption ratio. Lower limb muscle strength is fundamental to gait stability and propulsion. The development of muscle strength directly affects children’s stability, gait propulsion, and step smoothness during walking (19). In this study, measurements of muscle strength revealed differences in gait control across different age groups of children. Joint power reveals the energy conversion and transfer mechanisms at the joints during the gait process. The analysis of joint power helps to understand how the joints collaborate efficiently to promote movement efficiency (20). Additionally, variations in joint power reflect the energy consumption and recovery mechanisms of gait, which are crucial to gait stability and efficiency. he energy-absorption ratio—defined here as the relation between negative (energy-absorbing) and positive (energy-generating) joint power—serves as an index of transient energy storage and return during stance and push-off phases and thus informs both energetic economy and dynamic stability (21). Variability in these measures across age groups provides mechanistic insight into shifts in muscle–tendon contributions, inter-segmental energy transfer, and compensatory control strategies during gait maturation.

By synthesizing coordination metrics (via PCA), local dynamic stability (via LyE), and targeted biomechanical measures (muscle strength, joint power, energy-absorption ratio), the study adopts a multidimensional framework to characterize gait maturity. This integrative approach enhances the capacity to delineate developmental differences in neuromuscular control, dynamic stability, and energetic efficiency across preschool age cohorts. Whereas prior investigations have frequently targeted clinical or atypical populations—such as children with autism spectrum disorder (3, 22) or cerebral palsy (23, 24) —or concentrated on spatiotemporal descriptors in typically developing samples (25), comprehensive biomechanical evaluations of healthy preschool cohorts remain limited. To address this gap, the present study systematically examines joint-angle and joint-moment coordination in 3–5-year-old children using infrared motion capture coupled with Anybody 7.4 musculoskeletal simulation. In addition, age-related differences in muscle strength, mean joint power, and the energy-absorption ratio during gait will be quantified to identify salient features of motor development across this critical window.

**Figure 1:**
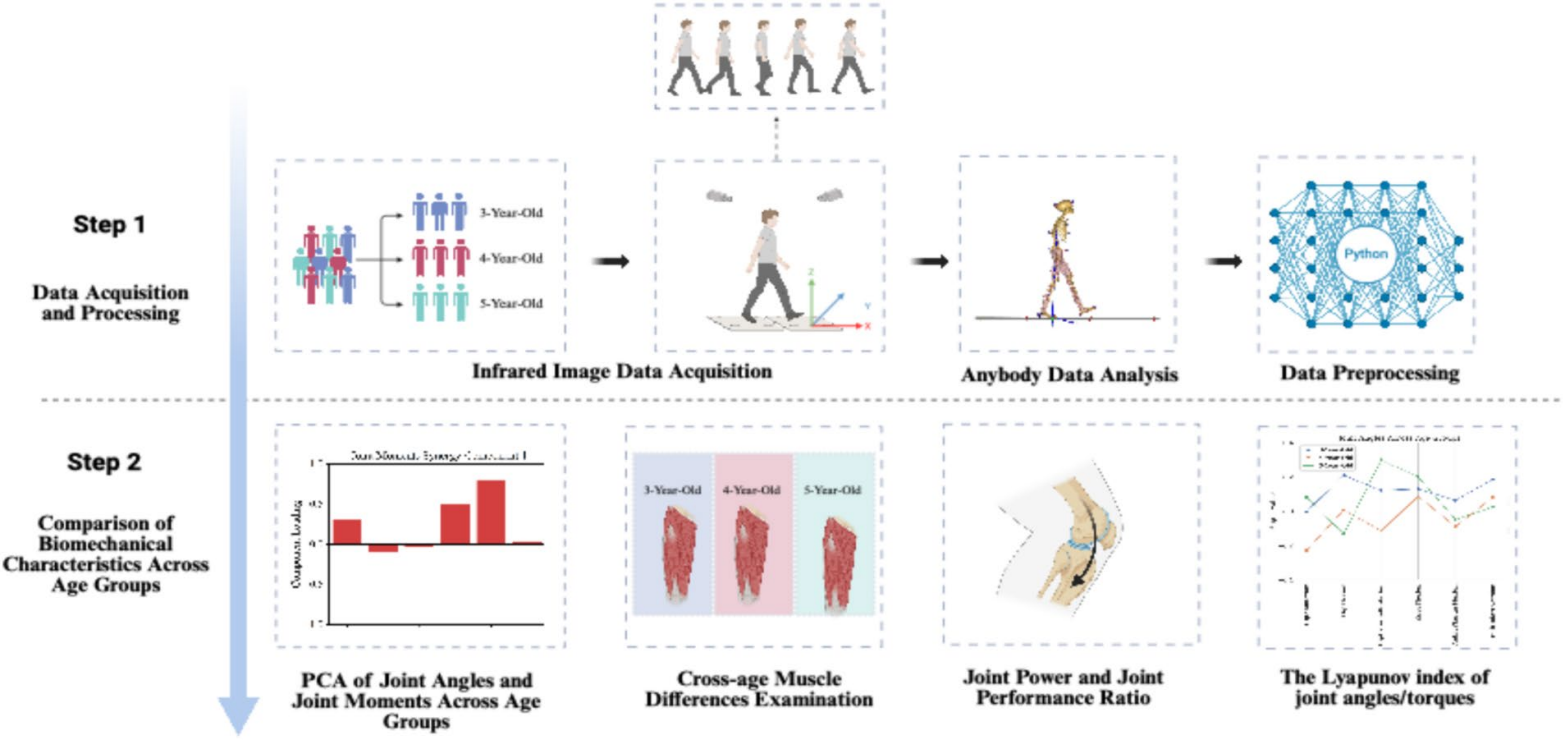
Study design created with BioRender.com.

Through this study, we aim to provide new insights into the gait development of healthy preschool children and offer a scientific basis for early interventions to help identify children with abnormal gait development, thereby laying a foundation for improving their future motor abilities. The full research process is illustrated in Figure 1.

## 2 Participants and methods

### 2.1 Participants

This study recruited 3–5 years old children from a private kindergarten in Renqiu City who met the test criteria and voluntarily participated in the experiment. With the assistance of teachers, detailed information about the subjects was obtained, including socioeconomic background, physical activity levels, and any potential confounding factors that might affect gait development, such as parental education level, and frequency of physical activity. Children with limited mobility, limb or organ diseases, or unstable health conditions were excluded. In total, 56 children completed the infrared data measurement. Before the infrared data measurement, markers were applied to the subjects according to the method shown in Table 1. The subjects were then asked to walk normally across the force plate to collect infrared data. During data processing, data with marker displacement and abnormal values were excluded. Their basic information is shown in Table 2. This study was approved by the Ethics Committee of the Faculty of Psychology at Beijing Normal University, with the approval number: 201910210061. During the study, informed consent forms were distributed to the parents of the participants, and the testing procedures were explained in detail to the teachers.

**Table 1:** The placement information of marker points.

Placement of marker points	Number
C7 Vertebra	1
Left/Right Acromion	2
Left/Right Anterior Superior Iliac Spine	2
Midpoint between Left and Right Posterior Superior Iliac Spines	1
Left/Right Greater Trochanter	2
Left/Right Mid-Thigh	2
Left/Right Lateral Tibial Condyle	2
Left /Right Fibular Head	2
Left/Right Mid-Calf	2
Left/Right Lateral Malleolus	2
Left/Right Heel	2
Left/Right Fifth Metatarsal	2

**Table 2:** Subject information.

Group	Age (years)	Height (cm)	Weight (kg)
3-Year-old (*n* = 20)	3.40 ± 0.49	105 ± 5.40	17.95 ± 2.25
4-Year-old (*n* = 16)	4.31 ± 0.46	112.38 ± 3.52	20.50 ± 3.26
5-Year-old (*n* = 20)	5.60 ± 0.48	119.2 ± 6.49	23.42 ± 3.83

### 2.2 Methods

#### 2.2.1 Data collection

**Figure 2:**
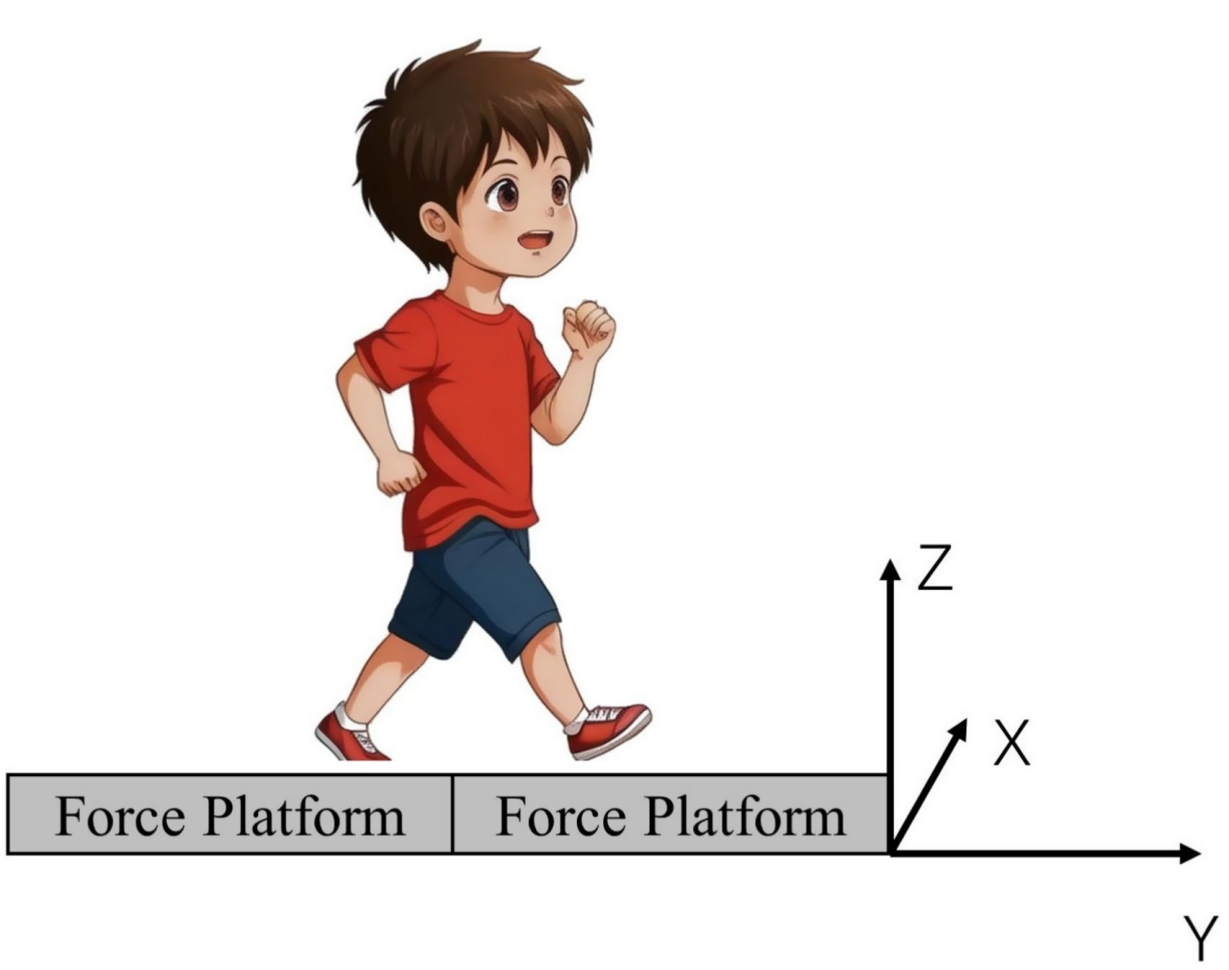
Force plate information.

Before collecting the infrared imaging data, measurements of key morphological indicators were taken for 56 children, including height, weight, head width, ankle-hip distance, knee width, leg length, hip depth, and pelvic width. Subsequently, the BTS SMART DX infrared optical motion capture analysis system was used, along with eight high-speed cameras and 22 reflective markers, to accurately capture the three-dimensional motion data of the subjects. Detailed information about the marker points is provided in Table 1. Simultaneously, the Kistler 3D force plate was used to collect dynamic data in real time during the subjects’ gait process, where the X-axis of the force plate points to the left, the Y-axis points forward, and the Z-axis points up ward (Figure 2).

Gait Analysis: The subjects first stood naturally with their feet parallel 3–5 meters away from Force Plate 1. Upon hearing the command, they walked normally across the force plate. In this study, the frame count at the moment the right foot contacts the force plate is defined as the starting point of the gait cycle, continuing until the moment the right foot leaves the force plate, thus constituting a complete gait cycle.

#### 2.2.2 Data analysis

By importing data collected from the BTS CAPTURE infrared capture software into Anybody 7.4, this study selected the complete cycle frame count of the children’s gait action when both feet were placed on two separate force plates. The lower limb gait model was then adjusted based on morphological data. Subsequently, through system parameter optimization, kinematic and inverse dynamics analysis, the study obtained data on the right lower limb joint angles, joint moments, and muscle Strengths of the children during one movement cycle. This study primarily analyzed the kinematic and dynamic characteristics of the supporting lower limb. The specific data collected included joint angles and moments for hip flexion, hip abduction, hip external rotation, knee flexion, ankle plantar flexion, and Sub Talar joint eversion of the supporting side; simultaneously, the collected muscle data encompassed the rectus femoris (RF), vastus medialis (VM), vastus lateralis (VL), biceps femoris (BF), semitendinosus (SED), semimembranosus (SEB), gastrocnemius (GAT), soleus (SL), tibialis anterior (TA), gluteus maximus (GMa), gluteus medius (GMe), and gluteus minimus (GMi) of the supporting side. Based on the measured data, further analysis yielded the following results: joint angle coordination patterns, joint moment coordination patterns, average muscle strengths, average joint power, joint energy absorption ratios, and Lyapunov indices. The specific calculation formulas are as follows:

(1) $$W=\left[\begin{array}{cccc} w_{11} & w_{12} & \cdots & w_{1p}\\ w_{21} & w_{22} & \cdots & w_{2p}\\ \vdots & \vdots & \ddots & \vdots \\ w_{m1} & w_{m2} & \cdots & w_{mp} \end{array}\right]$$

Principal Component Analysis (PCA) is a powerful tool for dimensionality reduction that reveals the primary patterns or coordination patterns in the data by projecting the original data into a new coordinate system. In PCA, the principal component matrix W represents the coordination patterns. Each column represents a principal component, which is a linear combination of the original data. In Equation 1, $w_{mp}$ is an element of matrix W that indicates the weight of the m -th feature in the original data on the p -th principal component. Here, m is the number of original features (i.e., the number of rows), and p is the number of principal components (i.e., the number of columns).

(2) $$X_{transformed}=X\cdot W$$

The original data X is transformed into the principal component space by multiplying it by the principal component matrix W . The representation of the data in the principal component space can be expressed using Equation 2. Here, X is the original data matrix, where rows represent samples and columns represent features (e.g., joint moments or angles). W is the principal component matrix. $X_{\mathrm{transformed}}$ is the data matrix in the principal component space, representing the projection of the data onto the principal component coordinate system.

$$\lambda_{i}=\frac{Var\left(PC_{i}\right)}{Total\;Var}$$

(3) $$CumulativeVariance_{k}=\overset{k}{\underset{i=1}{\sum }}\;\lambda_{i}$$

In this paper, we use Equation 3 to analyze the cumulative variance ratios during the PCA across different ages, thereby revealing the main structure and patterns of the data. In Equation 3, $\lambda_{i}$ represents the proportion of explained variance for the i -th principal component. $\mathrm{Var}\left(PC_{i}\right)$ is the variance of the i -th principal component, and TotalVar is the total variance of all principal components. ${\mathrm{Cumulative Variance}}_{k}$ represents the cumulative proportion of explained variance for the first k principal components.

(4) P(t)=M(t)×ω(t)

Joint power is calculated using Equation 4, where P(t) is the joint power at time t , and M(t) is the joint moment at time t , and ω(t) is the joint angular velocity at time t .

(5) $$EnergyAbsorptionRatio=\frac{\underset{P\left(t\right)<0}{\sum }\;P\left(t\right)}{\underset{P\left(t\right)>0}{\sum }\;P\left(t\right)}$$

The energy absorption ratio for each joint is obtained using Equation 5, where $\underset{P\left(t\right)<0}{\sum }\;P\left(t\right)$ is the sum of all negative power, representing the energy absorbed by the joint, and $\underset{P\left(t\right)>0}{\sum }\;P\left(t\right)$ is the sum of all positive power, representing the energy output by the joint.

(6) $$ℶ=\frac{1}{N}\overset{N}{\underset{i=1}{\sum }}\ln\left(\frac{d\left(t_{i}+\tau \right)}{d\left(t_{i}\right)}\right)$$

In this study, the Lyapunov index is calculated using Equation 6. Here, ℶ represents the Lyapunov index, which measures the degree of divergence of the system’s trajectory in phase space; a higher value indicates a stronger chaos in the system. N represents the number of iterations, which corresponds to the summation count in the equation. $d\left(t_{i}\right)$ is the initial distance between two time points $t_{i}$ , representing the difference in joint moments or angles at time $t_{i}$ in the time series, $d\left(t_{i}+\tau \right)$ is the distance between these two points in phase space after a delay of time τ , representing the difference in joint moments or angles at time $t_{i}+\tau$ . ln denotes the natural logarithm, used to quantify the proportion of distance change in the system, avoiding the impact of negative values on the calculation.

### 2.3 Statistical analysis

This study used Python 3 for data processing and standardized the dynamic data of muscle strength and joint moments for all participants by weight to eliminate the impact of individual weight differences on the results. In the analysis of significant differences in muscle strength among children of different ages, normally distributed data were analyzed using one-way ANOVA, while non-normally distributed data were analyzed using the Kruskal-Wallis H test. The number of coordination patterns for joint angles and joint moments was determined through PCA, using the cumulative variance index to decide on the coordination patterns, defining the optimal number of patterns as when the cumulative variance reaches over 95%.

## 3 Result

**Figure 3:**
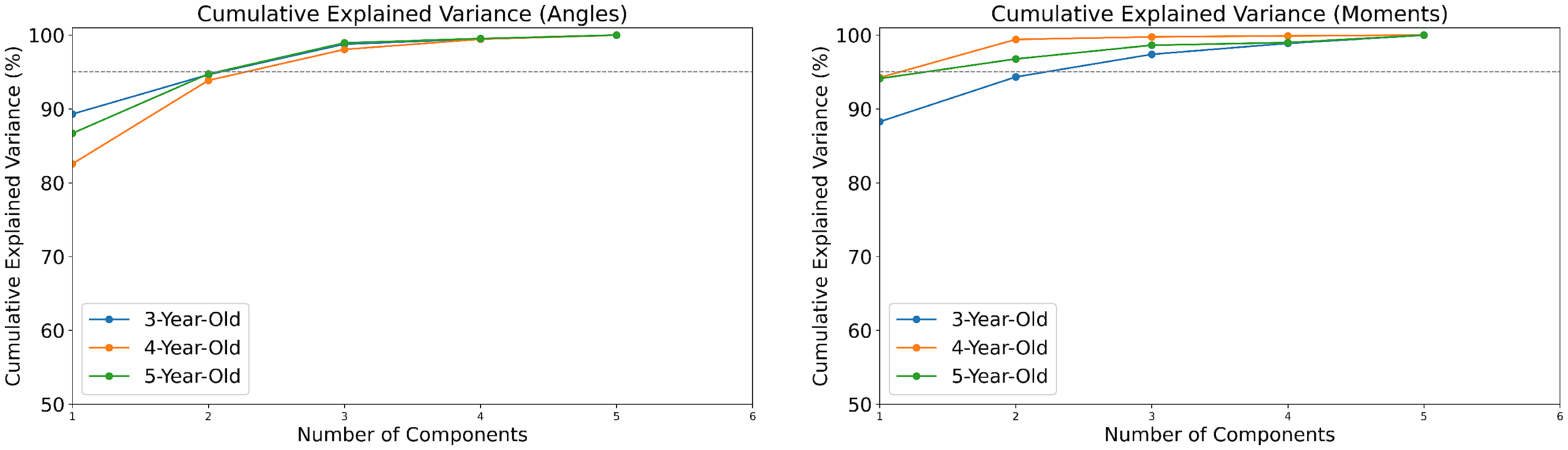
The cumulative variance index of joint angles/torques in children aged 3–5.

In analyzing the coordination patterns of joint angles and joint moments during a complete movement cycle for children of different ages, we employed PCA and determined the number of coordination patterns based on the cumulative variance ratio. According to Figure 3, children aged 3–5 exhibited three coordination patterns for joint angles during the completion of a movement cycle; for joint moments, 3-year-old children had three patterns, while 4- and 5-year-old children had two patterns each.

### 3.1 Coordination patterns of joint angles and joint moments

#### 3.1.1 Coordination patterns of joint angles

**Figure 4:**
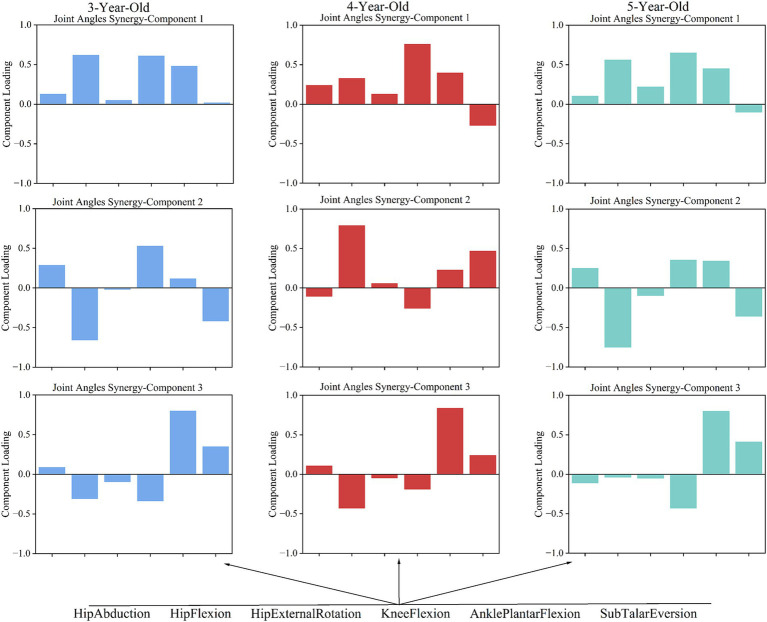
The coordination patterns of joint angles in children aged 3–5.

In analyzing the gait characteristics of the supporting phase of the lower limb joints during walking in children of different ages, it was found that 3-, 4-, and 5-year-old children utilized three main coordination patterns for joint angles during the completion of the movement, with specific characteristics as follows:

In 3-year-olds, Pattern 1 is dominated by hip and knee flexion, with ankle plantar flexion also contributing (Figure 4). This pattern suggests that 3-year-old children primarily focus on bending the hip and knee to stabilize the body, while the ankle plantar flexion contributes to pushing off the ground to assist in forward movement. Pattern 2 mainly involves hip and knee flexion, with minimal hip rotation and ankle movement. This pattern highlights a simplified coordination strategy where the focus is more on hip and knee flexion, with less emphasis on ankle control, reflecting the early stage of motor development. Pattern 3 shows ankle plantar flexion as dominant, with some hip and knee flexion. This pattern indicates that the ankle is playing a larger role in propulsion, while hip and knee flexion are used to a lesser degree, demonstrating early ankle control development. In 4-year-olds, Pattern 1 highlights knee flexion, followed by ankle and hip flexion. This pattern reflects a more developed coordination strategy where knee flexion plays a key role in shock absorption and stability, with the hip and ankle contributing to propulsion. Pattern 2 emphasizes hip flexion, with some ankle and hip rotation. This pattern represents an increased involvement of the hip and its rotation. Pattern 3 highlights ankle plantar flexion with some hip flexion. This pattern shows a more refined control of the ankle, with hip flexion serving as a secondary contributor. In 5-year-olds, Pattern 1 highlights knee flexion, followed by hip and ankle movements. Pattern 2 focuses on hip flexion, with ankle movement. This pattern shows a greater involvement of the hip and ankle, indicating better stability and control in the lower limbs. Pattern 3 shows ankle plantar flexion as dominant, with some hip rotation and knee flexion. This pattern reflects hip rotation and knee flexion contributing to a more coordinated gait.

#### 3.1.2 Coordination patterns of joint moments

**Figure 5:**
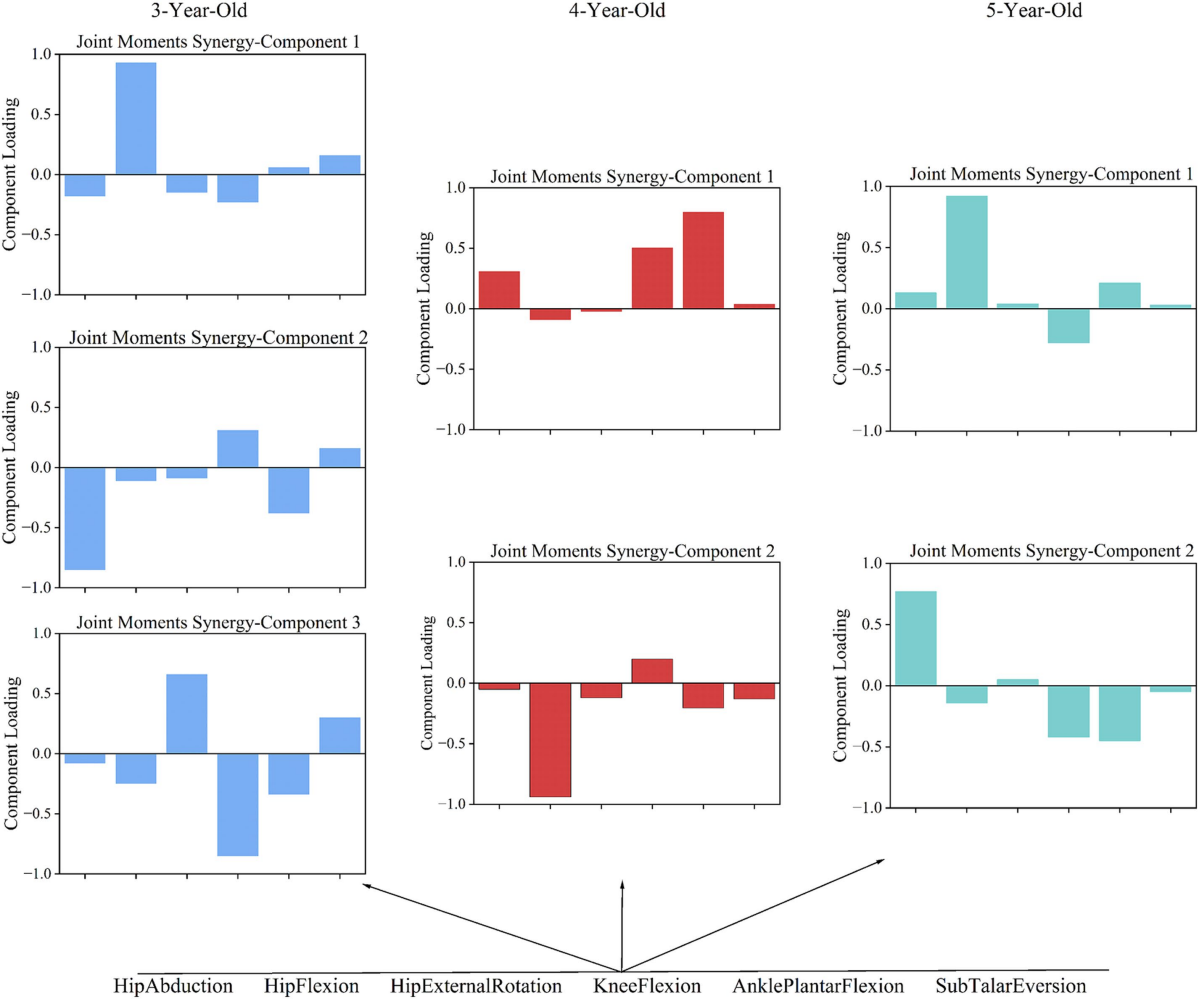
The coordination patterns of joint torque in children aged 3–5.

Torque coordination analysis revealed 3 patterns in 3-year-olds, and 2 patterns in 4- and 5-year-olds. In 3-year-olds, Pattern 1 is dominated by hip flexion torque, with minor hip rotation and knee flexion torques (Figure 5). This pattern indicates an emphasis on hip flexion, with less contribution from knee and hip rotation. Pattern 2 mainly involves hip abduction torque, with knee and ankle torques contributing. This pattern shows a larger role for the hip in stabilizing the pelvis and assisting with lateral movements, while the knee and ankle provide additional torque. Pattern 3 highlights knee flexion torque, with some ankle torque. This pattern focuses on knee flexion, suggesting that the knee plays a more prominent role in stability and shock absorption. In 4-year-olds, Pattern 1 is dominated by ankle torque, followed by knee torque. This pattern reflects a shift toward greater control at the ankle and knee, signaling more refined coordination for stability and propulsion. Pattern 2 highlights hip flexion torque, with ankle and knee torque contributions. This pattern emphasizes the involvement of the hip flexion torque. In 5-year-olds, Pattern 1 is dominated by hip flexion torque, followed by ankle torque. This pattern represents ankle torque assisting in propulsion. Pattern 2 highlights hip abduction torque, with knee torque. This pattern indicates a more complex lateral movement strategy involving hip abduction and knee torque for stability.

### 3.2 Muscle strength and joint dynamics results

#### 3.2.1 Muscle strength

As shown in Table 3, primary muscle strength of the supporting limb was evaluated during movement execution in children aged 3, 4, and 5 years. The principal results are summarized below.

**Table 3:** Muscle strength.

Muscle	Muscle strength (N/kg)			Effect size			Confidence interval		
	3yo	4yo	5yo	3 vs. 4	4 vs. 5	3 vs. 5	3 vs. 4	4 vs. 5	3 vs. 5
RF	2.86 ± 2.17	1.65 ± 1.21^a^	2.14 ± 1.18^c^	0.69	0.41	0.41	(0.01, 1.37)	(−0.25，1.07)	(−0.21, 1.04)
VM	0.56 ± 0.33	0.68 ± 0.52	0.71 ± 0.34^b,c^	−0.38	0.07	0.45	(−0.38, 0.94)	(−0.59, 0.73)	(−0.18, 1.08)
VL	3.83 ± 1.94	2.91 ± 1.22^a^	1.86 ± 0.91^b,c^	0.57	0.98	1.30	(−0.10, 1.24)	(0.28, 1.67)	(0.62, 1.98)
BF	3.54 ± 1.28	11.29 ± 4.1^a^	7.82 ± 3.81^b,c^	2.55	0.88	1.51	(1.67, 3.43)	(0.19, 1.56)	(0.80, 2.21)
ST	2.57 ± 1.65	10.42 ± 8.72^a^	6.30 ± 5.10^b,c^	1.25	0.58	0.98	(0.53, 1.97)	(−0.09, 1.25)	(0.33, 1.64)
SM	0.46 ± 0.39	2.71 ± 1.39^a^	2.94 ± 2.43^b,c^	2.20	0.12	1.43	(1.37, 3.04)	(−0.54, 0.77)	(0.73, 2.12)
GA	8.69 ± 2.04	11.47 ± 3.17^a^	7.95 ± 4.01^c^	1.04	0.97	0.23	(0.34, 1.74)	(0.28, 1.67)	(−0.39, 0.85)
SOL	3.83 ± 2.42	3.24 ± 1.11	2.07 ± 0.80^b,c^	0.31	1.21	0.98	(−0.35, 0.97)	(0.50, 1.92)	(0.32, 1.63)
TA	5.64 ± 3.88	4.52 ± 3.47^a^	9.39 ± 3.69^b,c^	0.30	1.36	0.99	(−0.36, 0.97)	(0.63, 2.09)	(0.33, 1.65)
GMa	0.55 ± 0.28	1.35 ± 1.29^a^	1.52 ± 1.27^b^	0.86	0.13	1.05	(0.17, 1.54)	(−0.53, 0.79)	(0.39, 1.72)
GMe	1.19 ± 0.41	1.87 ± 0.58^a^	1.61 ± 0.72^b,c^	1.35	0.40	0.72	(0.63, 2.08)	(−0.27, 1.06)	(0.08, 1.36)
GMi	0.90 ± 0.23	0.57 ± 0.31^a^	0.82 ± 0.47^b,c^	1.21	0.63	0.22	(0.49, 1.92)	(−0.05, 1.30)	(−0.41, 0.84)

“a” indicates a significant difference between 3 and 4 years old (*p* < 0.05), “b” indicates a significant difference between 3 and 5 years old (*p* < 0.05), and “c” indicates a significant difference between 4 and 5 years old (*p* < 0.05). RF stands for Rectus Femoris, VM stands for Vastus Medialis, VL stands for Vastus Lateralis, BF stands for Biceps Femoris, SED stands for Semitendinosus, SEB stands for Semimembranosus, GAT stands for Gastrocnemius, SL stands for Soleus, TA stands for Tibialis Anterior, GMa stands for Gluteus Maximus, GMe stands for Gluteus Medius, and GMi stands for Gluteus Minimus.

RF strength measured 2.86 ± 2.17 N/kg in 3-year-olds, decreased to 1.65 ± 1.21 N/kg at 4 years, and increased to 2.14 ± 1.18 N/kg at 5 years; the value at 5 years was significantly greater than at 4 years (*p* < 0.05).

VM strength rose from 0.56 ± 0.33 N/kg in 3-year-olds to 0.68 ± 0.52 N/kg in 4-year-olds (difference not statistically significant), and further to 0.71 ± 0.34 N/kg at 5 years; the 5-year value was significantly higher than those at both 3 and 4 years (*p* < 0.05).

VL strength was 3.83 ± 1.94 N/kg in 3-year-olds, and declined significantly to 2.91 ± 1.22 N/kg in 4-year-olds and to 1.86 ± 0.91 N/kg in 5-year-olds (*p* < 0.05 for the declines).

BF strength increased markedly from 3.54 ± 1.28 N/kg in 3-year-olds to 11.29 ± 4.10 N/kg in 4-year-olds (*p* < 0.05). At 5 years BF strength decreased to 7.82 ± 3.81 N/kg but remained significantly higher than in 3-year-olds (*p* < 0.05).

SED strength rose to 10.42 ± 8.72 N/kg in 4-year-olds from 2.57 ± 1.65 N/kg in 3-year-olds (*p* < 0.05), and declined to 6.30 ± 5.10 N/kg at 5 years while remaining significantly greater than the 3-year value (*p* < 0.05).

SEB strength increased to 2.71 ± 1.39 N/kg in 4-year-olds, significantly different from 0.46 ± 0.39 N/kg in 3-year-olds; at 5 years SEB strength further rose to 2.94 ± 2.43 N/kg and remained significantly higher than in 3-year-olds (*p* < 0.05).

GAT strength was 8.69 ± 2.04 N/kg in 3-year-olds, increased to 11.47 ± 3.17 N/kg in 4-year-olds (*p* < 0.05 vs. 3 years), and decreased to 7.95 ± 4.01 N/kg at 5 years; the 5-year value did not differ significantly from the 3-year value but was weaker than the 4-year value (*p* < 0.05).

SL strength was 3.83 ± 2.42 N/kg in 3-year-olds, decreased to 3.24 ± 1.11 N/kg in 4-year-olds and further to 2.07 ± 0.80 N/kg in 5-year-olds; the decline at 5 years was significant relative to the younger ages (*p* < 0.05).

TA strength increased significantly to 9.39 ± 3.69 N/kg in 5-year-olds, compared with 5.64 ± 3.88 N/kg in 3-year-olds and 4.52 ± 3.47 N/kg in 4-year-olds (*p* < 0.05).

GMa strength was 0.55 ± 0.28 N/kg in 3-year-olds, rose to 1.35 ± 1.29 N/kg in 4-year-olds (*p* < 0.05 vs. 3 years), and further increased to 1.52 ± 1.27 N/kg at 5 years (*p* < 0.05 vs. both younger groups).

GMe strength measured 1.19 ± 0.41 N/kg in 3-year-olds, increased to 1.87 ± 0.58 N/kg in 4-year-olds (*p* < 0.05), and decreased to 1.61 ± 0.72 N/kg at 5 years while remaining significantly higher than in 3-year-olds (*p* < 0.05).

Based on the results in Table 3, the observed age-related changes in primary supporting-leg muscle strength were non-linear and muscle-specific. Certain muscles (e.g., BF, SED, GAT) peaked at 4 years and then declined by 5 years, whereas others (e.g., VM, TA, GMa) exhibited progressive increases with age; conversely, VL and SL showed progressive declines. These patterns indicate heterogeneous maturation trajectories among the muscle groups that contribute to stance-phase control during gait.

#### 3.2.2 Joint power and joint energy absorption ratio

According to Table 4, hip abduction power was 0.02 W/kg for 3- and 5-year-olds, and slightly lower at 0.01 W/ kg for 4-year-olds. Hip flexion power was highest in 4-year-olds (0.19 W/ kg), significantly higher than in 3- and 5-year-olds. Hip external rotation power was −0.01 W/ kg for 3-year-olds, increasing slightly to 0.001 W/ kg for 4-year-olds, and decreasing to −0.003 W/ kg for 5-year-olds. Knee flexion power increased with age: 0.02 W/ kg for 3-year-olds, 0.09 W/ kg for 4-year-olds, and 0.023 W/ kg for 5-year-olds. Ankle plantar flexion power increased from 0.02 W/ kg at age 3 to 0.06 W/ kg at age 5. Sub Talar Eversion power was negative in all groups: −0.01 W/ kg for 3-year-olds, and −0.02 W/ kg for 4- and 5-year-olds.

**Table 4:** Average joint power.

Joint	Average joint power (W/kg)		
	3-Year-old	4-Year-old	5-Year-old
Hip Abduction	0.02	0.01	0.02
Hip Flexion	0.06	0.19	0.07
Hip External Rotation	−0.01	0.001	−0.003
Knee Flexion	0.02	0.09	0.02
Ankle Plantar Flexion	0.02	0.05	0.06
Sub Talar Eversion	−0.01	−0.02	−0.02

Regarding the joint energy absorption ratio (Table 5), Hip abduction energy absorption was negative: −0.93 at age 3, −0.89 at age 4, and −0.88 at age 5. Hip flexion energy absorption was −0.78 at age 3, −0.80 at age 4, and −0.76 at age 5. Hip external rotation energy absorption fluctuated: −1.05 at age 3, −0.90 at age 4, and −1.06 at age 5. Knee flexion absorption was −0.87 at age 4 and −0.86 at age 5, both higher than −0.74 at age 3. Ankle plantar flexion absorption was consistent at −0.94 for 3- and 4-year-olds, dropping to −0.87 at age 5. Sub Talar Eversion absorption was negative in all groups, with little variation: −1.10 at age 3, −1.09 at age 4, and −1.12 at age 5.

**Table 5:** Joint energy absorption ratio.

Joint	Joint energy absorption ratio		
	3-Year-old	4-Year-old	5-Year-old
Hip Abduction	−0.93	−0.89	−0.88
Hip Flexion	−0.78	−0.80	−0.76
Hip External Rotation	−1.05	−0.90	−1.06
Knee Flexion	−0.74	−0.87	−0.86
Ankle Plantar Flexion	−0.94	−0.94	−0.87
Sub Talar Eversion	−1.10	−1.09	−1.12

Collectively, these findings indicate that hip flexion power attains a maximum at age four, whereas ankle plantar-flexion power increases progressively with age. Subtalar joint mechanics are consistently absorptive across the examined ages. Energy-absorption ratios are predominantly negative across joints and ages; however, hip external-rotation absorption displayed greater variability across the three cohorts.

### 3.3 Lyapunov index

The analysis of the Lyapunov exponent for joint angles indicated unique dynamic stability characteristics among children of different age groups (Table 6). This transition from joint angle coordination to Lyapunov exponent analysis highlights the shift from kinematic coordination to dynamic stability, showing how movement coordination evolves into dynamic stability control as children age. In 3-year-olds, hip abduction showed very high stability (−0.003), while hip flexion (0.210) and external rotation (0.120) had weaker stability. Knee flexion (0.130) and ankle plantar flexion (0.063) showed good stability, but Sub Talar Eversion was weaker (0.186). For 4-year-olds, hip abduction (−0.229), flexion (0.007), and external rotation (−0.114) showed higher stability, while knee flexion (0.086), ankle plantar flexion (−0.090), and Sub Talar Eversion (0.081) also had good stability. In 5-year-olds, hip abduction (0.081) had moderate stability, while hip flexion (−0.134) had higher stability. Hip external rotation (0.298) showed poorer stability. Knee flexion (0.199), ankle plantar flexion (−0.049), and Sub Talar Eversion (0.024) demonstrated moderate to high stability.

**Table 6:** The Lyapunov index of joint angles/torques.

Joint		3-Year-old	4-Year-old	5-Year-old
Angles	Hip Abduction	−0.003	−0.229	0.081
	Hip Flexion	0.210	0.007	−0.134
	Hip External Rotation	0.120	−0.114	0.298
	Knee Flexion	0.130	0.086	0.199
	Ankle Plantar Flexion	0.063	−0.090	−0.049
	Sub Talar Eversion	0.186	0.081	0.024
Torques	Hip Abduction	−0.062	−0.046	−0.019
	Hip Flexion	−0.056	−0.043	0.009
	Hip External Rotation	−0.078	0.015	0.002
	Knee Flexion	−0.038	0.031	−0.057
	Ankle Plantar Flexion	0.614	−0.037	0.049
	Sub Talar Eversion	0.030	−0.069	−0.206

In the analysis of the Lyapunov exponent for joint moments (Table 6), significant differences in dynamic stability were also observed among children of different age groups. This transition from joint moment coordination to Lyapunov analysis emphasizes the increasing complexity of dynamic control in older children, showing how joint torque coordination evolves into more stable and complex dynamic behaviors. In 3-year-olds, hip abduction (−0.062), flexion (−0.056), and external rotation (−0.078) had high stability. Knee flexion (−0.039), ankle plantar flexion (0.061), and Sub Talar Eversion (0.030) showed good stability. In 4-year-olds, hip abduction (−0.046) and flexion (−0.043) showed strong stability. Hip external rotation (0.015) had moderate stability, while knee flexion (0.031), ankle plantarflexion (−0.037), and Sub Talar Eversion (−0.069) showed variable stability. For 5-year-olds, hip abduction (−0.019), flexion (0.009), and external rotation (0.002) showed higher stability. Knee flexion (−0.058) and ankle plantarflexion (0.050) demonstrated high stability, while Sub Talar Eversion (−0.206) showed very high stability. As children age, their joint torque stability improves. The fluctuations in 5-year-olds’ indices suggest more complex gait control, balancing short-term stability with flexibility, reflecting gait maturation.

## 4 Discussion

### 4.1 Analysis of coordinated patterns in children of different ages

Gait requires coordinated joint actions to handle movement complexity (10, 11). The central nervous system simplifies motor processes, improving control efficiency (26–28). The neuromuscular system plays a key role in coordinating joints and muscles (29). This study uses PCA to analyze the kinematic and torque patterns in the lower limbs of children aged 3–5 during the gait support phase, uncovering gait characteristics and developmental patterns.

The study shows that 3-year-olds use complex multi-joint coordination in gait, but their strategies are immature, leading to instability. In joint angle coordination pattern 1, hip and knee flexion show high synergy, while the ankle contributes to gait movement. This indicates that children use hip and knee movements along with the ankle to propel forward, a pattern also important for 4- and 5-year-olds (Figure 4). Research suggests ankle maturation occurs around age 4 (9). The key transition at age 4 is reflected in the increasing involvement of the ankle joint in coordination patterns, suggesting that this age marks the shift toward the ankle assuming a more independent role in gait.

In the gait support phase, 3-year-olds show high synergy in knee and hip flexion in joint angle coordination pattern 1, while hip flexion and knee extension dominate in torque patterns 1 and 3 (Figure 4). This suggests that 3-year-olds may need counteracting torques at the knee to maintain balance during gait, indicating they have not mastered joint angle and torque coordination fully. However, at age 4, there is a notable shift in the coordination between the hip and knee joints, marking a transition from reliance on compensatory strategies to more integrated joint control.

In joint angle coordination pattern 2, there is a significant synergy between hip extension and knee flexion (Figure 4). Sub Talar joint eversion enhances stability and cushioning during gait, but its significant eversion (−0.423) may lead to instability due to insufficient coordination with other movements. This synergy also shows similarities in the joint coordination patterns of 4- and 5-year-olds. At age 4, this synergy between the hip and knee joints begins to stabilize, indicating that gait control is gradually shifting toward more coordinated and stable strategies.

In joint angle coordination pattern 3, ankle plantar flexion is dominant for 3-year-olds. This matches findings that ankle maturation occurs around age 4, while hip and knee maturation occurs around age 7 (9). Joint torque pattern 2 for 3-year-olds shows significant negative participation from hip flexion, potentially leading to uneven torque distribution and affecting gait stability (Figure 4). The negative involvement of hip flexor torque at age 3 suggests this is a characteristic of early gait control, with uneven torque distribution possibly leading to instability in gait. The negative participation of the hip joint during dynamic center of mass transfer may be a typical feature of early gait control in children, with uneven torque distribution possibly leading to instability in gait control.

In 4- and 5-year-olds, torque coordination characteristics gradually decrease with age. Observations of joint angle coordination patterns across ages show similarities in gait actions, especially in pattern 2. The transition at age 4 is further emphasized by the increased coordination between the hip and knee joints, especially with the involvement of ankle plantarflexion, marking a shift toward more balanced gait control. In 4-year-olds, hip flexion (0.797) dominates pattern 2, coordinating with Sub Talar Eversion and ankle plantar flexion. This suggests that 4-year-olds still rely on the hip joint heavily and have insufficient multi-joint coordination in complex gait tasks, primarily using the hip joint to drive movement (Figure 4). This reliance on the hip joint at age 4 illustrates the critical transitional nature of this stage, where children are still working on integrating distal joints into a more balanced coordination strategy.

In 5-year-olds, joint coordination pattern 2 shows hip extension (−0.751) dominating, with knee flexion, ankle plantar flexion, and Sub Talar inversion (−0.355) working together. This indicates a more complex gait control strategy, allowing better handling of complex movements (Figure 4). By age 5, this transition is reflected in a more coordinated joint control strategy, where children begin to integrate the full lower limb into their motor control system. In joint coordination pattern 3, children’s coordination becomes more efficient with age. Coordination in this pattern is generally similar across all three age groups. However, while 3- and 4-year-olds have ankle plantar flexion dominating, 5-year-olds show more efficient coordination. This pattern shows clear joint angle coordination with ankle plantar flexion, knee extension, and Sub Talar Eversion forming joint angle pattern 3. While the other two age groups also have dominant ankle plantar flexion, the involvement of other joints varies, complicating their coordination.

Joint torque coordination patterns show greater variability with age compared to coordination patterns. According to Figure 5, we find that 3-year-olds use three main joint torque patterns to complete actions, while 4- and 5-year-olds use two. This suggests that as children age, their ability to coordinate joint torques improves. Interestingly, the three patterns seen in 3-year-olds can be encompassed by the two patterns in 5-year-olds. In contrast, 4-year-olds show more variability in their joint torque patterns compared to the other age groups. This variability at age 4 reflects the transitional nature of motor control during this period, where children are refining their strategies but still exhibit a range of movement patterns.

In joint torque pattern 1, 3-year-olds show a hip flexion-dominant pattern. This is similar to the pattern in 5-year-olds, but 5-year-olds also coordinate hip flexion with knee extension and ankle plantar flexion, showing more complex and mature coordination. In joint torque pattern 2, hip extension dominates for 4-year-olds, with greater ankle joint participation compared to 3-year-olds. This suggests that 4-year-olds are starting to focus on coordinating the ankle and hip joints. In joint torque patterns 2 and 3 for 3-year-olds, actions mainly involve hip adduction and knee extension. These patterns suggest that their overall coordination ability is relatively singular, making it hard to compensate for deficiencies in other joints, resulting in less stability. From Figure 5, we observe that in joint torque pattern 1 for 4-year-olds, ankle plantar flexion torque significantly increases, and torque distribution becomes more balanced between hip and knee joints, indicating greater reliance on ankle strength. In gait progression, the use of distal lower limb strength becomes clearer, allowing better management of center of gravity changes during gait, which enhances stability and efficiency. In joint torque pattern 2, 5-year-olds show increased hip abduction torque, with a balanced distribution between knee and hip joint torques. Minimal contribution from other joints indicates effective use of distal lower limb strength while maintaining stability.

Overall, from ages 3–5, children show more complex and diverse joint coordination strategies in gait. 3-year-olds have a more singular pattern, primarily relying on hip and knee flexion for gait. Meanwhile, 4-year-olds show greater variability in coordination patterns, indicating a critical stage in motor development. The key transition at age 4 is marked by the increasing integration of distal joints, and the shift toward more balanced coordination strategies. By age 5, children effectively coordinate multiple joint strengths during gait and demonstrate flexible strategies for different tasks. This aligns with Blandine Bril’s research, which suggests that 5- to 6-year-olds can maintain balance using only their leg muscles during walking (30). Despite improvements in gait control, their immaturity and dependency indicate that further development and refinement in motor skills are needed.

### 4.2 Muscle strength and joint dynamics characteristics

This section analyzes the differences in the dynamics of the ankle, knee, and hip joints and related muscle strength in 3, 4, and 5-year-olds during gait. The aim is to explore the characteristic differences in motor control strategies across different age groups. Previous studies have shown that joint dynamics during gait are crucial for gait completion. For example, Salam M’s research indicates that hip extension torque significantly influences the ground reaction force during the early phase of gait (31).

In 3-year-olds, hip abduction power is low (0.02 W/Kg), possibly due to insufficient strength in the abductor muscles (e.g., GMe and Gmi) (Tables 3, 4). This low power reflects a lack of control ability in hip abduction during gait in 3-year-olds. They mainly rely on high energy absorption (energy absorption ratio of −0.93) to maintain gait stability (Table 5). This reliance on energy absorption rather than propulsion highlights the developmental stage at age 3, where stability is achieved through passive mechanisms, marking it as a foundational phase of motor development. By age 4, hip abduction power further decreases to 0.01, although this power reduction occurs. However, children may partially compensate for the lack of abductor strength by enhancing GMe strength (Tables 3, 4). This shows transitional characteristics in gait control. This shift in muscle strength suggests a transitional phase at age 4, where children begin to rely more on active muscle control rather than passive mechanisms. By age 5, hip abduction power rises to 0.02 W/Kg, and the relative stability of GMe strength indicates that their gait control strategies have matured. Through more efficient muscle coordination, they achieve more stable hip abduction movements and propulsion in gait (Tables 3, 4). Previous research also indicates that increased hip abductor strength contributes to enhanced gait speed (32).

In 3-year-olds, hip flexion power is low (0.06 W/Kg), mainly relying on high energy absorption to maintain gait stability (Tables 4, 5). This characteristic may lead to insufficient hip flexion strength, affecting propulsion and rhythm in gait. This increase in hip flexion power at age 4 marks the onset of a critical transition in motor control, where the ability to generate propulsion becomes more pronounced. As they age, hip flexion power in 4-year-olds significantly increases to 0.19 W/Kg, indicating enhanced propulsion strength. At this stage, gait control is transitional, displaying some gait fluctuations. By age 5 (Tables 3–5), hip flexion power slightly declines to 0.07 W/Kg, but the strength of the RF increases. This indicates that 5-year-olds can achieve stable and efficient hip flexion movements through more effective muscle coordination and energy utilization.

The study finds that hip external rotation power remains at low levels across all age groups (Tables 3–5). This indicates that the main function of hip external rotation in gait is to maintain stability rather than produce propulsion. For 3-year-olds, hip external rotation strength mainly relies on the GMe. Although hip external rotation power increases at age 4, energy utilization at this age tends to balance out. By age 5, the strength of the GMe and GMi stabilizes. The energy absorption ratio of hip external rotation indicates that gait control strategies are gradually maturing. This gradual improvement, particularly noticeable by age 4, underscores the importance of this age as a transition point where stability and muscle coordination begin to show clear improvements.

The knee joint plays a crucial role in generating propulsion during gait. Previous studies show that the knee extensors control the knee during the support phase of gait, generating necessary propulsion (33, 34). Decreased knee flexor strength may lead to shorter stride length at self-selected walking speeds (35). This study finds that knee flexion power significantly increases at age 4, closely related to the significant strengthening of the BF and SED (Tables 3–5). This indicates that children in this age group rely more on knee flexion movements for gait propulsion. However, 3-year-olds show low knee flexion power, indicating insufficient propulsion in gait. In 5-year-olds, knee flexion power slightly declines, but improved coordination of knee muscles enhances energy absorption ratios. This indicates the gradual maturation and stability of gait control strategies.

The ankle joint plays a more significant role in generating propulsion. Research shows that ankle dorsiflexion and plantar flexion directly affect gait balance and speed (36). Contraction of the ankle flexors can produce forward propulsion and the propulsion generated accounts for approximately 67% of the total joint power during the stance phase of gait (37–39). This study finds that with age, the dynamics of the ankle joint during gait varies: As age increases, ankle plantar flexion power gradually increases, indicating a growing demand for propulsion during gait. The GAT strength in 3-year-olds is relatively high, but it slightly decreases in 5-year-olds as they age (Table 3). However, through stable output from the SL, energy absorption ratios improve, indicating that energy utilization in ankle plantar flexion becomes increasingly effective (Tables 3–5). The output of gait propulsion becomes more stable and mature. In contrast, Sub Talar joint eversion shows negative power across all age groups, indicating its primary role in stability rather than propulsion during gait. Overall, children exhibit a high energy absorption ratio at this joint, indicating its greater role in stability during gait. This is especially crucial in regulating stability during unsteady gait.

This chapter primarily reveals the differences in the dynamics of the hip, knee, and ankle joints and muscle strength among 3, 4, and 5-year-olds during gait. The results indicate that the gait stability of 3-year-olds mainly relies on high energy absorption. Four-year-old show transitional characteristics in gait control, with significant increases in knee flexion power and muscle strength. These transitional characteristics are particularly indicative of the developmental milestone at age 4, where the ability to generate propulsion and improve joint coordination becomes more apparent. By age 5, gait control matures, achieving stable propulsion through more efficient muscle coordination. Especially, 4-year-olds are in a critical period of motor development, with notable transitional gait characteristics.

### 4.3 Analysis of the stability of joint angles and joint torques

To further analyze the differences in gait control strategies among children of different ages, this study incorporates the Lyapunov index of joint angles and torques as a measure of dynamic stability in gait. The Lyapunov index reflects the sensitivity of movements to small disturbances, with higher values indicating greater instability.

During walking, hip external rotation (40) and abduction (41, 42) affect the kinematic and dynamic characteristics of the knee joint in normal gait. Based on the Lyapunov index (Table 6), it was found that as age increases, the Lyapunov indices for hip abduction and external rotation decrease during the transition from ages 3 to 4, indicating that the stability of these joints improves during this stage. This transition at age 4 corresponds to a key developmental stage, as evidenced by the significant reduction in angular variability and the improvement in joint coordination, marking the shift toward more coordinated and stable movements. By age 5, we observed an increase in the Lyapunov indices for both hip external rotation and abduction angles, as well as a rise in hip flexion torque, which seems contradictory to the conclusion of enhanced stability. However, by examining the coordination patterns of joint angles and torques, we found that 5-year-olds exhibit greater flexibility and complexity in movement adjustment, enabling them to cope with larger gait fluctuations without compromising overall stability (Figures 4, 5). Specifically, although the Lyapunov index is higher at age 5, they may maintain gait stability through enhanced neuromuscular coordination. Previous studies have shown that hip flexion plays an important role in completing the gait process (43, 44).

Based on Table 6, we found that the stability of hip flexion angle increases with age, indicating that the control capability of hip flexion improves as children grow, leading to more mature gait control. In the Lyapunov index changes for hip flexion torque, we found that it increases with age, suggesting a transition from rigid stability to more flexible, dynamic gait control strategies. The knee joint is an important driving joint in human walking, contributing to forward propulsion (33), adjusting stride length (35) and influencing lower limb coordination (45). Analyzing the Lyapunov index of knee flexion in different children, we found a significant drop from 3 to 4 years, indicating increased stability in 4-year-olds, though this also correlates with greater rigidity, which may reduce knee flexibility. This marked reduction in variability at age 4 further emphasizes the transition toward greater stability, aligning with the milestone of improved motor control. By age 5, the Lyapunov index for knee flexion angle shows a significant increase, reflecting greater dynamic variability as children adapt to more complex task demands in gait control. As children grow, their motor skills improve, and they may attempt more challenging movements or gait adjustments, leading to greater angular variability and flexibility. Observing the Lyapunov index for knee flexion torque, we noted a rise at age 4, followed by a decline at age 5, indicating fluctuations as children explore control strategies for applying torque (Table 6). By age 5, the decline in the torque Lyapunov index indicates enhanced stability in applying torque. As they age, children develop finer muscle control, allowing for more stable torque application; even with increased angular variability, their control over force becomes more precise, reducing excessive torque fluctuations. As one of the three major joints in gait, ankle dorsiflexion plays a crucial role in generating propulsion (37, 39) and controlling body posture (46, 47). In this study, the Lyapunov index for preschool children’s ankle dorsiflexion during gait cycles showed a decrease followed by a slight increase from ages 3–5, while the torque Lyapunov index initially dropped sharply and then slightly rose. This change indicates that children’s gait control abilities are gradually improving, particularly with significant stability at age 4. By age 5, although angular variability slightly increases, this may be due to children’s enhanced flexibility to adapt to more complex gait changes and movement demands. Simultaneously, torque control becomes more refined and stable, indicating their ability to effectively control the forces applied during gait, leading to gradual maturation. Previous research shows that the degree of pronation in the Sub Talar joint correlates with stability in gait (48). The Lyapunov index for Sub Talar pronation angle and torque control gradually decreases with age, indicating improved stability in gait control over the years (Table 6). Notably, at age 5, the angle Lyapunov index is very low (close to 0), while the torque Lyapunov index drops significantly into the negative range, indicating that children achieve high stability in movement control at this joint and exhibit refined and stable force control. Overall, the gait control system in children at this age is developing toward greater maturity and stability.

## 5 Conclusion

This study examined gait development in 3- to 5-year-olds children by analyzing joint kinematics, torque coordination, and gait stability during the support phase. The key findings are:

At age 3, children show immature gait control characterized by poor stability and simple coordination strategies, mainly relying on hip and knee synergy with limited ankle involvement.

At age 4, gait control enters a transitional phase. Increased variability and greater ankle participation suggest that children begin developing more dynamic and adaptive movement strategies.

By age 5, although neuromuscular development is still ongoing, children demonstrate more refined multi-joint coordination and efficient muscle output, enabling more stable and flexible gait patterns compared to younger ages.

In summary, gait control in children from ages 3–5 progresses from simple to more coordinated patterns, with age 4 being a key transition. These findings can help design early intervention programs, especially for children with delayed motor development or abnormal gait. Additionally, this knowledge can be applied in sports education and pediatric screening to identify children in need of intervention. Future studies could explore how to incorporate these findings into clinical and educational practices to enhance children’s motor skills and health.

### 5.1 Limitations

This study utilized a sample from a single preschool located in a specific geographic region, which may introduce regional biases into the findings. As the sample was drawn from one location, the results may not fully represent the gait development patterns of children from different regions, considering potential differences in cultural backgrounds, environmental factors, and educational resources. However, despite these limitations, the sample provides valuable insights into gait development within a particular context, offering a foundational basis for future research. To enhance the generalizability of the findings, future studies should consider incorporating a more diverse sample from various regions and types of early childhood education institutions, which would help validate and extend the current results.

## Data Availability

The raw data supporting the conclusions of this article will be made available by the authors, without undue reservation.
